# Experiences and needs of people with haematological cancers during the COVID‐19 pandemic: A qualitative study

**DOI:** 10.1002/pon.5819

**Published:** 2021-09-09

**Authors:** Nienke Zomerdijk, Michelle Jongenelis, Eva Yuen, Jane Turner, Kathryn Huntley, Andrew Smith, Megan McIntosh, Camille E. Short

**Affiliations:** ^1^ Melbourne School of Psychological Sciences University of Melbourne Melbourne Victoria Australia; ^2^ Victorian Comprehensive Cancer Centre Alliance Melbourne Victoria Australia; ^3^ Melbourne School of Psychological Sciences Melbourne Centre for Behaviour Change University of Melbourne Melbourne Victoria Australia; ^4^ Centre for Quality and Patient Safety Research–Monash Health Partnership Institute for Health Transformation Deakin University Melbourne Victoria Australia; ^5^ School of Nursing and Midwifery Faculty of Health Deakin University Melbourne Victoria Australia; ^6^ School of Psychology and Public Health Department of Psychology and Counselling La Trobe University Bundoora Victoria Australia; ^7^ Psycho‐Oncology Research Unit Olivia Newton‐John Cancer Wellness and Research Centre Austin Health Heidelberg Victoria Australia; ^8^ Faculty of Medicine University of Queensland Brisbane Queensland Australia; ^9^ Cancer Care Services Royal Brisbane and Women's Hospital Brisbane Queensland Australia; ^10^ Leukaemia Foundation Brisbane Queensland Australia; ^11^ Faculty of Health and Medical Sciences University of Adelaide South Australia Australia; ^12^ School of Health Sciences University of Melbourne Melbourne Victoria Australia

**Keywords:** cancer, COVID‐19 pandemic, haematology, oncology, psycho‐oncology, qualitative research, supportive care

## Abstract

**Objective:**

Haematological cancer patients are particularly vulnerable to the effects of COVID‐19. In addition to being immunocompromised, pandemic‐related travel restrictions have impacted access to treatments and overseas stem cell donations for patients requiring transplantation. Given this vulnerability, people with haematological cancers are at risk of experiencing heightened distress during the pandemic. This study aimed to explore haematological cancer patients' experiences and needs.

**Methods:**

Twenty‐four Australian haematological cancer patients completed semi‐structured interviews exploring their concerns and worries during the pandemic, impact of pandemic on management of disease, access to information and support, lifestyle changes, and attitudes towards emerging models of healthcare during the pandemic. Interview transcripts were thematically analysed.

**Results:**

Four themes reflecting the experiences of haematological cancer patients during the pandemic were identified: ‘Fears about contracting COVID‐19' (behaviour changes to protect health, impact on daily routine and habits, annoyance at dismissive attitude of others toward COVID‐19); ‘Reduced sense of connection and support’ (reduced social support and access to external support services); ‘New challenges’ (increased financial hardship, worsened health), and; ‘Underlying system and communication issues' (access to trusted information, satisfaction/dissatisfaction with care, navigating telehealth). Participants expressed a need for improved access to support services and trusted information.

**Conclusions:**

The findings emphasise the additional challenges experienced by haematological cancer patients during the COVID‐19 pandemic and their impact on daily life. Results point to the importance of validation of increased distress during periods of uncertainty; reinforcing recommendations about high‐quality sources of information; and facilitating access to support services when face‐to‐face care is limited.

## 1 BACKGROUND

Haematological cancers are a unique and heterogenous group of diseases that require specialised attention and care.^1^ Despite some similarities between patients diagnosed with these diseases and solid cancers, treatment regimens for haematological cancers are comparatively long, aggressive, and require ongoing maintenance. Furthermore, the highly immunosuppressive status induced by these treatments and the diseases itself necessitates precautions, such as self‐isolation, to minimise risk of acquiring infections that can cause life‐threatening complications.^2^

During the COVID‐19 pandemic, the challenges faced by people with haematological cancers have both been illuminated and exacerbated.^3^ The increased vulnerability to infections and viruses among those with haematological cancers has caused significant concern about a heightened risk of COVID‐19 related morbidity and mortality, and the available data seem to support these concerns. A recent meta‐analysis comprising 3377 patients from three continents reported a mortality risk of 34% among adults with haematological cancer and COVID‐19.^4^ This figure is substantially higher than the mortality rate reported in patients with solid cancers (25%),^5^ emphasising the vulnerability of this subgroup and the importance of engagement in behavioural strategies that limit risk of infection (e.g., physical distancing, enhanced hygiene practices).

Access to treatments and overseas stem cell donations is another challenge faced by haematological cancer patients during the COVID‐19 pandemic. In Australia, more than 80% of haematological cancer patients requiring haematopoietic stem cell transplantation (HSCT) receive stem cells from international volunteer donors.^6^ Travel restrictions during the pandemic have reduced access to overseas donors, creating significant logistical challenges and psychological distress for those planned to undergo HSCT.^7^

A growing body of literature attests to the negative psychological impacts of the COVID‐19 pandemic on people living with cancer, including increased fear of disease progression, anxiety, and depression.^8^, ^9^, ^10^, ^11^ Qualitative findings suggest that the challenges created by the pandemic (e.g., treatment interruptions, fears of contracting COVID‐19, reduced access to support from healthcare providers and family, and financial hardship) have contributed to heightened distress among cancer patients.^10^, ^12^ There have also been growing concerns about observed reductions in the number of cancer patients accessing care during the pandemic.^13^, ^14^, ^15^, ^16^ The precise reasons for this are unclear but may relate to patients' fear of acquiring COVID‐19 in a clinical setting, limited capacity to use telehealth tools, and concerns about wasting the clinician's time.^17^, ^18^ The consequences of delays in accessing care may be far reaching.

Understanding the psychological impact of the pandemic can help inform the development of new models of care that address concerns and unmet needs during and beyond this pandemic. While existing literature highlights the psychological concerns of patients with solid cancers during the pandemic, given the unique challenges and needs faced by people with haematological cancers, more focused, in‐depth studies are needed to identify the unmet psychological and supportive care needs in this population. In‐depth understanding of these needs in haematological cancer patients has the potential to inform the delivery of optimal care for this patient group. Accordingly, this study aimed to advance understanding of haematological cancer patients' experiences, concerns, and unmet needs during the COVID‐19 pandemic.

## 2 METHODS

This study forms part of a broader project examining the psychosocial impact of the COVID‐19 pandemic on people with haematological cancers. Study approval was granted by the Human Research Ethics Committee of the University of Melbourne (Ref: 2057125.1).

### 2.1 Participants and recruitment

Eligible participants were adults aged ≥18 years who currently have, or previously had, a confirmed diagnosis of haematological cancer and had sufficient English language skills to participate without an interpreter. Participants who resided outside of Australia were excluded. Participants responded to a study advertisement distributed via email and/or social media platforms by an established national community group (Leukaemia Foundation), a professional working group (Victorian COVID‐19 Cancer Network), and a clinical trial group (Australasian Leukaemia & Lymphoma Group). Potential participants were provided with a link to an online information sheet, consent form, and survey.

After completion of the survey, participants were invited to participate in an interview to obtain a better understanding of their experiences and needs during the pandemic. Of the 394 participants who completed the survey, 125 (32%) expressed interest in completing an interview. Purposive sampling techniques were used to select interview participants who (1) had received a haematological cancer diagnosis since 2004 and (2) had an appointment scheduled during the pandemic. Additionally, we aimed to ensure there was equal representation of participants with high levels of psychological distress according to the self‐reported Kessler 10 item Psychological Distress scale^19^ (score ≥30 on the Kessler 10) and low levels of distress (score ≤20 on the Kessler 10). Purposively selected participants were contacted by researchers NZ and EY via email to schedule an interview. Of the 40 participants emailed, 24 chose to participate, representing a response rate of 60%. Verbal and written informed consent were obtained prior to the interviews. In line with Braun and Clarke's reflexive thematic analysis approach,^20^ data saturation was not the intention of recruitment and analysis. Rather, the intention was to broadly and meaningfully explore patient experiences and care needs.

### 2.2 Data collection

Interviews were conducted by authors NZ and EY (who both hold PhDs in psycho‐oncology and are trained in qualitative research methods) between August and November 2020. A semi‐structured interview guide was used (see Table S1), which incorporated broad and open‐ended questions created by the authors based on a review of clinically relevant issues identified in the COVID‐19 literature and our previous research,^21^ clinical experience, and consultation with haematology healthcare professionals. A panel of haematology healthcare professionals, psycho‐oncological academics, and haematology patients assessed the appropriateness of the interview guide, and all agreed that the questions were understandable and relevant. Topics covered by the questions included experiences during the pandemic, impact of the pandemic on management of disease, perceived adequacy of information and support, lifestyle changes, and perceived benefits and challenges of using telehealth tools. Interviews took place via telephone and were on average 29 min in length (range 18–49).

### 2.3 Data analysis

Interviews were audio‐recorded using a voice recording device and transcribed verbatim. Reflexive thematic analysis of the transcripts was guided by Braun and Clarke's six‐stage method.^20^, ^22^ All transcribed interviews were read and checked against the original recordings to ensure accuracy and familiarisation with the content (step one). To prevent potential analysis bias, the primary coder (MM) was blinded to the distress level of the participants. Familiarisation and initial coding of a subset of the transcripts was conducted by authors MM and NZ. The authors (MM, NZ) independently coded the data and met to determine whether they agreed with the codes identified. After discrepancies were resolved through discussion and consensus was achieved, coding on remaining transcripts was performed by MM (step two), and codes were categorised ‘patient experiences’ and ‘system and communication issues’ (step three). These categories were then refined into themes and formally defined (steps four and five) and subsequently summarised with extracts from the transcripts (step six).

## 3 RESULTS

### 3.1 Sample characteristics

A total of 24 haematological cancer patients, with a median age of 57 years (range 38–81) completed interviews between August and November 2020; 46% had high levels of psychological distress (score ≥30 on the Kessler 10^19^). The ratio of men to women was even (50%) and most were married (79%). The most frequently reported haematological cancers were Leukaemia (37%), Lymphoma (25%), and Myeloma (21%). Further sample details are reported in Table 1.

**TABLE 1:** Sample characteristics (*n* = 24)

Characteristic	*n* (%)
Gender	
Male	12 (50)
Female	12 (50)
Age, mean ± SD (range)	57.3 ± 10.6 (38–72)
Marital status	
Married or Defacto	19 (79.2)
Single, divorced, separated, or widowed	5 (20.8)
Location	
Major city	16 (66.7)
Regional	8 (33.3)
Education	
Secondary school or below	3 (12.5)
Trade or another certificate	10 (41.7)
University degree	11 (45.8)
Current employment status	
Employed	9 (37.5)
Unemployed	6 (25)
Not in labour force/Retired	9 (37.5)
Years since diagnosis, mean ± SD (range)	
Primary diagnosis	2.8 ± 3.6 (0–15)
Leukaemia	9 (37.5)
Lymphoma	6 (25)
Myeloma	5 (20.8)
Other haematological cancers	4 (16.7)
Disease status	
Not yet started active treatment	1 (4.2)
Undergoing curative treatment	4 (16.7)
Completed treatment and in remission	3 (12.5)
Ongoing treatment to manage disease	12 (50)
Other^a^	4 (16.7)

^a^ Other disease status include completed transplant but remission status unknown, ‘watch and wait’ management strategy, treatment to manage graft‐versus‐host‐disease.

### 3.2 Overview of themes

Analysis of the qualitative data resulted in four overarching themes: “Fears about contracting COVID‐19”, “Sense of connection”, “New challenges”, and “System and communication issues”. Each theme contained sub‐themes. Illustrative quotes are presented in text.

### 3.3 Fears about contracting COVID‐19

#### 3.3.1 Behaviour changes to protect health

The fear of contracting COVID‐19 and the life‐threatening impact it would have on their health was pervasive for participants, leading to feelings of anxiety, acute fear, and distress.

- ‘I was pretty fearful of dying actually, that was, I think that's the bottom line… I had a couple of days where it definitely, um, linked back into when I was first diagnosed. Um, and the fear level was, was similar and I had a little bit of a, what I call a meltdown.’ (P13, Lymphoma, treatment ongoing)

Participants reported self‐implemented behaviour changes such as wearing masks and gloves, working from home, keeping children at home, washing hands, and disinfecting household items. These were all attempts to protect their health and to cope with the fear associated with COVID‐19.

- ‘I have stayed home most of the time. When I do go out, I wear gloves and a mask. I come home and wash my hands. I go out only if I have to.’ (P2, Autoimmune haemolytic anaemia, treatment completed)

#### 3.3.2 Impact on daily routine and habits

Many participants described the impact of the physical distancing measures on their daily routines and habits. This ranged from avoidance of routine habits such as grocery shopping and commuting on public transport to the cancellation of social events. Some participants also noted consuming more alcohol and eating more than usual since the COVID‐19 outbreak, which they attributed to spending more time at home. Participants expressed profound disappointment over reduced opportunities for physical activity.

- ‘I've always done best exercising in a group setting and we lost access to that as well. So it was quite hard to um, keep up that motivation and that sort of exercise while we were locked down.’ (P9, Leukaemia, treatment ongoing)
- ‘Um, but, I, my vice has been food, so I've put on 10 kilos. Um, because, you know, I've tried to occupy myself. I haven't been able to go out or, you know, just…’ (P7, Leukaemia, treatment ongoing)

#### 3.3.3 Annoyance at dismissive attitudes of others toward COVID‐19

Many expressed their annoyance towards those who they viewed as not taking the pandemic seriously. Participants described their disappointment toward others, including family members, friends, and members of the general public, who did not take precautions to help protect vulnerable immunocompromised persons.

- ‘It's very frustrating, because we've got a beautiful big family, they just didn't self‐isolate, ever… Even when they were supposed to, because, oh its family, we love each other, we can't spread it.’ (P7, Leukaemia, treatment ongoing)

### 3.4 Reduced sense of connection and support

#### 3.4.1 Reduced social support

Participants were aware of their vulnerability and recognised the importance of minimising their risk of COVID‐19 infection. Many distanced themselves from important social connections such as close family and friends and stayed home as much as possible. Although crucial, these protective measures exacerbated pre‐existing concerns of loneliness for participants who had previously undergone treatments that involved lengthy hospitalisations.

- ‘I think it's just… It's very isolating to have this type of cancer… and just made multiply worse by COVID.’ (P17, Leukaemia, treatment ongoing)

No‐visitor policies in hospitals meant participants could not be accompanied during their treatment. This was challenging for participants who missed out on the support and comfort provided by their caregivers.

- ‘I guess my last five months of treatment, my wife couldn't come with me… because of what's happening… That was disappointing because, you know, you like a bit of support when you're in there… It would've been nice to have her in there.’ (P15, Lymphoma, treatment ongoing)

### 3.5 Reduced access to external support services

Participants found it challenging to access psychosocial support during the pandemic due to the cancellation or reorganisation of many support services. While some maintained contact with previously‐accessed support services, others were unaware if services were being continued virtually, or reported difficulty attending them due to their density limits or capacity restrictions. This resulted in a loss of support.

- ‘They did tell me there was an online support group, that was very minimal. There's minimal placings, so to try and get a spot was difficult… all of them are booked out… That's how important the support groups are to us, you know, it's that important.’ (P16, Myeloma, treatment ongoing)

### 3.6 New challenges

#### 3.6.1 Increased financial hardship

Increased financial hardship was experienced by some participants, especially by those who noted that they were currently unemployed due to illness and/or their caregiver was unable to maintain paid employment either because they lost their job or chose not to work in order to mitigate the risk of contracting COVID‐19 and transmitting the virus to the patient at home. In these instances, participants expressed concerns about being unable to take care of living expenses and family needs.

- ‘We didn't have the money because my partner's work had been shut down. I'd been out waiting income protection, we were pretty… We had no money really.’ (P18, Lymphoma, undergoing curative treatment)
- ‘My son, uh, was at uni and working and he stopped working, and um, and my daughter stopped working as well, so, just to reduce the risk.’ (P14, Myelodysplastic syndrome, in remission)

#### 3.6.2 Worsened health

The pandemic brought new challenges for managing health. Several participants discussed experiencing increased anxiety and depression, often in relation to feelings of loneliness and fears of contracting COVID‐19.

- ‘I've had, I think at times extreme anxiety attacks. Largely because of my concern for my family.’ (P1, Leukaemia, treatment ongoing)
- ‘It's the loneliness… I've always been a loner, but this is something different… And I think it's just, um, there's a lot of fear in me… There are a lot of nights where I just go to bed and I just think, you know, ‘Let me just have a nice big stroke and not wake up at all.’ (P17, Leukaemia, treatment ongoing)

Additionally, some participants experienced an increase in their cancer‐related symptoms, such as bone pain and fatigue, which they attributed to reduced activity.

- ‘I got more bone pain because I noticed I stopped, you know, moving as much.’ (P7, Leukaemia, treatment ongoing)

### 3.7 System and communication issues

#### 3.7.1 Access to trusted information

Very few participants discussed receiving specific advice from their clinician regarding their treatment or precautions to minimise their risk of infection. Many participants sought their own information from government‐related websites but found it difficult to obtain information regarding the risks and impacts of COVID‐19, leading to uncertainty about how to protect their health and wellbeing. This uncertainty was compounded by rapid changes in information from trusted sources about the impacts of COVID‐19 and the effectiveness of strategies to contain it.

- ‘I kept in tune with the numbers, where the risks were, could we go out? Could I go out? Could my wife go out? Where should we go shopping? Should we not go shopping? All those kinds of things.’ (P2, Autoimmune haemolytic anaemia, treatment completed)

The lack of clear communication was especially noted by participants who had to travel interstate to receive treatment.

- ‘I mean initially we were chasing our own information, we had to try and find out what the situation was via the Tasmanian government website. When we got to Melbourne, we didn't know whether we'd be tested or quarantined or what, because the only thing we could find out was via the news and what was on the Internet.’ (P12, Myeloma, treatment ongoing)

#### 3.7.2 Satisfaction and dissatisfaction with care

Many participants were satisfied with the changes made to protect their health. The sense that healthcare professionals were ‘doing the best they can’ was commonly shared by participants. Those who were on clinical trials found their trial nurses to be particularly proficient in continuing care, treatment, and providing information.

- ‘My team has been fantastic. They have gone, you know, beyond the call of duty I suppose to try and assist and make things happen.’ (P9, Leukaemia, treatment ongoing)

Unfortunately, participants also expressed various dissatisfactions with their healthcare during the pandemic. Some experienced difficulties communicating with facilities that were not familiar with haematology, such as emergency departments or local clinics. Several participants also expressed concerns when hospital staff appeared to not follow guidelines, such as wearing personal‐protective equipment, or when the COVID‐19 clinics were visible from cancer clinics.

- ‘I go to hospital every four weeks because I have to have angiograms. No one wore masks. No one necessarily wore gloves.’ (P4, Leukaemia, treatment to manage graft‐versus‐host‐disease)

#### 3.7.3 Navigating telehealth

COVID‐19 brought significant changes to healthcare delivery. Most participants felt that telehealth saved them time and costs associated with face‐to‐face appointments. They considered it an acceptable alternative; a view held particularly by those who had pre‐existing relationships with healthcare professionals. However, navigating telehealth was challenging at times, with many perceiving it as less personal. While video calls were the preferred modality for appointments since it could allow patients to visually see their doctor, participants reported that it was rarely used or offered by their healthcare provider.

- ‘Telehealth is more convenient because you don't have to get up, you don't have to go and park, you don't have to sit in the waiting room and then only have a really short visit, which if you're not feeling well, I suppose, it's much more convenient.’ (P3, Myeloma, treatment ongoing)
- ‘It became a bit impersonal and yeah, you just felt more like a number… It just didn't feel proper, sort of thing. You'd get used to going in there and having that interaction and talking to your doctor and if anything came up you could do it on the spot, ask questions. Then in a phone call, just, things were all a bit rushed and yeah, I've often gone, ‘Oh shoot, I didn't ask this or I didn't ask that.’ (P6, Leukaemia, treatment ongoing)

## 4 DISCUSSION

This study aimed to advance understanding of haematological cancer patient experiences and unmet needs during the COVID‐19 pandemic using an in‐depth qualitative research method. Fear of contracting COVID‐19 emerged as a strong concern for haematological cancer patients. Participants' awareness of their vulnerability to COVID‐19 and its life‐threatening impact on their health was pervasive and led to feelings of anxiety, acute fear, and distress. This influenced behaviour changes to cope with the fear of contracting COVID‐19, such as distancing themselves from family and friends and disinfecting household items, similar to studies conducted with solid cancer patients during the pandemic.^10^, ^12^

Participants commonly described instances where supportive care services had been rescheduled, cancelled, or changed to online modalities. Many participants reported difficulty accessing services, with many not informed of services being continued virtually. These patients reported falling through the cracks of the systems and reported losing an important source of wellbeing and sense of connection from support services they had previously accessed. Our findings shed light on previous quantitative reports of reductions in cancer patients accessing care during the pandemic.^13^ This could relate to strict stay‐at‐home‐orders, border restrictions, mandated quarantine, and hospital visitor restrictions during the early stages of the pandemic in Australia, which severely disrupted non‐COVID‐19 related cancer care activities. Our results highlight the importance of clear communication between health professionals and/or cancer charities and haematological cancer patients regarding the availability of support services to ensure that psychosocial wellbeing is considered during crisis events such as pandemics. Moreover, a national planning effort led by the government in close collaboration with the health sector is needed to ensure that supportive cancer care services are maintained consistently across the country.

While haematological cancer patients continued to receive medical care via telehealth, this technology was perceived as less personal. Perhaps this is unsurprising since videoconferencing was preferred by participants yet reported to be rarely used or offered by healthcare professionals. These findings are consistent with recent data reporting that the majority of telehealth consultations occurred by telephone rather than videoconferencing.^23^, ^24^ The precise reasons for this are unclear but may relate to privacy and technical concerns, increased time needed for videoconferencing compared to telephone consultations, and lack of confidence using videoconferencing tools.^25^ Efforts to address these barriers and facilitate videoconferencing should be encouraged. Sansom‐Daly and Bradford^26^ argue that when done well, videoconferencing can be an intimate setting for psychosocial care with opportunities for assessing new cues (e.g., by seeing inside someone's home). Telephone only modalities on the other hand may particularly compromise effective interactions between healthcare professionals and patients since they limit access to facial cues, body language, and physical examination. Overall, our findings highlight an opportunity for patient‐centered care by offering videoconferencing tools in addition to telephone consultations. This is in accordance with Medicare guidelines,^27^ which recommend phone consultations be limited to no more than half of a practice's telehealth provisions. As described by Binder and colleagues,^28^ videoconferencing tools allow care teams to continue to provide quality care while minimising risk to haematological cancer patients.

This study found that haematological cancer patients experienced gaps in information during the COVID‐19 pandemic, which contributed to anxiety and fear. Very few participants described receiving advice from their clinicians, possibly due to the rapid changes in knowledge about COVID‐19 and lack of evidence to support early recommendations. This led to uncertainty about how their care would continue beyond the pandemic and the extent to which they should avoid routine habits and activities that pose an infection risk. Many participants relied on verified online sources but found it difficult to obtain information specifically targeted at people with haematological cancers. Participants, especially those who had to travel interstate to receive treatment, expressed the need for more detailed information about the risks and impacts of COVID‐19. With emerging evidence‐based guidelines, treating clinicians will more reliably be able to provide specific instructions. Discussions about strategies to avoid infection or risks of increased morbidity from the viral infection are important.^29^ Clearly, access to reliable information during pandemics is essential. During such crisis events, it is critical that both patients and their care teams are given access to the information and support they need.

Our findings also highlight the financial difficulties experienced by people with haematological cancers during the early stages of the COVID‐19 pandemic. While only a few participants were financially impacted by the pandemic, those who were described life‐changing implications. Participants who were unable to work or whose caregiver lost their job during the pandemic were concerned about being unable to afford basic needs. The theme underscores existing literature showing the financial burden can be substantial for people with haematological cancers due to the prolonged nature of treatments such as HSCT that can lead to extended time away from work.^30^, ^31^ The long‐term financial impacts of the pandemic on people with cancer are unclear and pose a risk of exacerbating distress which can lead to more serious problems, including depression, anxiety, and discontinued cancer care.^32^, ^33^ Further research with quantitative designs are needed to identify high‐risk subgroups who may benefit most from referral to targeted and freely available interventions, in order to mitigate the longer‐term financial impacts of the pandemic.

## 5 STUDY LIMITATIONS

First, an eligibility requirement that participants have sufficient English language skills is likely to have prevented the participation of individuals from culturally and linguistically diverse (CALD) backgrounds. Future research considering the needs of CALD individuals is critical. This is particularly important given the rapid uptake of telehealth consultations, which may be less suited to CALD patients and can exacerbate inequalities in cancer care.^6^, ^34^ Second, the experiences of participants who had not yet started active treatment were under‐represented in our sample. Further consideration of their unique experiences is important, particularly in light of the reported delays in accessing cancer care.^16^ Finally, our findings only illustrate the experiences of haematological cancer patients within Australia. The experiences of patients in other countries are likely to differ, owing to varying degrees of COVID‐19 restrictions and different healthcare systems. Alongside limitations, this study has several strengths. The recruitment method and purposive sampling technique used in this study resulted in a wide range of perspectives stemming from a representative balance of gender, haematological cancer types, treatment stages, and cities and regional areas. A further strength is the co‐development by and active participation of consumers and community organisations at all stages of the study.

## 6 CLINICAL IMPLICATIONS

The results of this study inform our recommendations to improve clinical care for haematological cancer patients. In the first instance, listening and acknowledging that some degree of distress is normal during this period of uncertainty (face‐to‐face when possible and via telehealth) can be helpful. For patients displaying moderate‐severe levels of distress, referral to low‐cost specialised psychosocial care can be considered. Second, we recommend offering videoconferencing in addition to audio‐only telephone consultations and increasing resources to facilitate these technologies. Third, it is critical that patients are provided with consistent information about the specific risks and impacts of COVID‐19. Ideally this would be delivered by treating clinicians. Fourth, maintaining support services during transitions of care as seamlessly as possible and clearly communicating how to access them is essential. Finally, we recommend promoting remote, home‐based lifestyle interventions to encourage engagement in positive lifestyle behaviours. These efforts combined can help to minimise disruptions to care for haematological cancer patients and ensure their psychosocial wellbeing is considered, which is particularly important during transitions from face‐to‐face to telehealth delivered care.

## 7 CONCLUSIONS

People with haematological cancers described several disruptions caused by the COVID‐19 pandemic. The pervasive fear of contracting COVID‐19, reduced opportunities for support, delayed care, information inadequacy, and increased financial hardship had a substantial impact on their physical and psychological wellbeing. The difficulties experienced by haematological cancer patients in accessing supportive care during the pandemic are particularly concerning given the increased uncertainty faced by this population because of the risks posed by COVID‐19, compounded by loneliness due to isolation. The reported challenges provide a unique perspective into how care for haematological cancer patients may be improved during and beyond the COVID‐19 pandemic.

## CONFLICT OF INTEREST STATEMENT

None to declare.

## AUTHOR CONTRIBUTIONS

All authors contributed to the study conception and design. Material preparation, data collection and analysis were performed by Nienke Zomerdijk, Michelle Jongenelis, Megan McIntosh, Eva Yuen, and Camille E. Short. The first draft of the manuscript was written by Nienke Zomerdijk and all authors commented on previous versions of the manuscript. All authors read and approved the final manuscript.

## Supporting information

Supplementary Material

## Data Availability

The data that support the findings of this study are available from the corresponding author upon reasonable request.
