# Supplementary material for: Experiences and needs of people with haematological cancers during the COVID‐19 pandemic: A qualitative study
Source: Psychooncology. 2021 Sep 9;31(3):416–24. doi: 10.1002/pon.5819 (PMC8646676; doi:10.1002/pon.5819)
Supplement: Supplementary file 1 — Supplementary Material [file PON-31-416-s001.docx]

**Table S1.** Interview guide

| Topic | Questions |
| --- | --- |
| Experiences during pandemic | Please tell me about your experience during the pandemic.  Can you tell me about any new concerns you have had during the pandemic?  How have these impacted you? |
| Management of disease | What impact, if any, has COVID-19 had on the management of your disease?  Has this worried you at all? |
| Information and support | Can you describe the way you received information during the COVID-19 pandemic?  How have you felt about the care provided by your healthcare team during the COVID-19 pandemic? |
| Telehealth tools | Did you utilise telehealth during the COVID-19 pandemic?  In your opinion, what were some of the advantages and challenges of using these tools?  Did you feel confident in using these mediums?  Were there any concerns you wanted your healthcare provider to know but didn’t feel comfortable raising in the telehealth consultation? |
| Lifestyle changes | Has your lifestyle changed during the COVID-19 pandemic? |
